# Correction to ‘Eco‐Microbiology: A Frugal‐Circular Framework for Biosafe, Low‐Cost Practical Microbiology in Secondary Education’

**DOI:** 10.1111/1751-7915.70487

**Published:** 2026-11-05

Amorim, L. , K. Timmis , B. da Silva Lopes , R. Ribeiro , and C. Santos . 2026. “Eco‐Microbiology: A Frugal‐Circular Framework for Biosafe, Low‐Cost Practical Microbiology in Secondary Education.” Microbial Biotechnology 19, no. 4: e70343. 10.1111/1751-7915.70343.PMC1308888441995289

The Author Contributions section is incomplete. The following contributions should be added:

*Betina da Silva Lopes: writing – review and editing*.

*Raquel Ribeiro: writing – review and editing*.

*Conceição Santos: validation, writing – review and editing*.

We apologize for this error.

